# Religious Attendance and Depressive Symptoms: The Mediating Role of Church-based Social Ties

**DOI:** 10.1093/geroni/igaf122.2712

**Published:** 2025-12-31

**Authors:** Mingui Gao, Hui Liu

**Affiliations:** Purdue University, West Lafayette, Indiana, United States; Purdue University, West Lafayette, Indiana, United States

**Keywords:** religious attendance, church-based social ties, depressive symptoms, mediation, HRS

## Abstract

This study used longitudinal data from the Health and Retirement Study (HRS) to examine whether church-based social ties (e.g., friendships, relatives in the congregation) mediate the relationship between religious attendance and depressive symptoms among older adults. Three hypotheses were tested: 1) Increased religious attendance is associated with fewer depressive symptoms, 2) Greater church-based social ties are linked to fewer depressive symptoms, and 3) The relationship between religious attendance and depressive symptoms is mediated by church-based social ties. Depressive symptoms were measured using an eight-item CES-D scale, and four waves of data (2010, 2012, 2014, and 2016) were analyzed using Ordinary Least Squares, Logit regressions, and Lagged Dependent Variable models. Findings suggest that more frequent religious attendance in 2010 was associated with fewer depressive symptoms in the shorter term (2014) and the longer term (2016). Moreover, church-based friendships mediated this relationship in 2016. These results provide insights into how religious participation impacts psychological well-being, which can inform interventions aimed at reducing depressive symptoms in older adults.

